# Comparative Study of Coagulation Profile and Haematological Parameters in Pregnancy-Induced Hypertension (PIH)

**DOI:** 10.7759/cureus.70529

**Published:** 2024-09-30

**Authors:** Garima S Agarwal, Anil K Agrawal

**Affiliations:** 1 Pathology, Jawaharlal Nehru Medical College, Datta Meghe Institute of Higher Education & Research, Wardha, IND

**Keywords:** coagulation profile, hematological parameters, hypercoagulable state, pre-eclampsia, pregnancy-induced hypertension, thrombocytopenia

## Abstract

Background

Pregnancy-induced hypertension (PIH) is a major cause of maternal and fetal morbidity and mortality. PIH, including gestational hypertension, pre-eclampsia, and eclampsia, often leads to significant alterations in coagulation profiles and haematological parameters, posing risks for thromboembolic and haemorrhagic complications. This study aimed to compare the coagulation and haematological parameters between women with PIH and normotensive pregnant women.

Methods

A cross-sectional observational study was conducted on 110 pregnant women, divided into two groups: 55 with PIH and 55 with normotensives. Coagulation markers such as prothrombin time (PT), activated partial thromboplastin time (aPTT), international normalised ratio (INR), and platelet count were measured. Bleeding and clotting times were also evaluated. Data were statistically analysed using student’s t-test and chi-square tests, with p ≤ 0.05 considered significant.

Results

Women with PIH exhibited significantly lower platelet counts, PT, aPTT, and INR values compared to the normotensive group (p < 0.0001). Thrombocytopenia and prolonged bleeding time were more common in the PIH group, suggesting an increased risk of both bleeding and clotting complications. Proteinuria was observed in 32 women (58.18%) in the PIH group, reflecting the severity of the disease. Additionally, the PIH group had markedly elevated blood pressure levels.

Conclusion

PIH is associated with significant coagulation and haematological abnormalities, including thrombocytopenia and a hypercoagulable state, which increase the risk of thromboembolic and hemorrhagic events. Routine monitoring of coagulation profiles and haematological parameters in PIH patients is essential for timely interventions and improving maternal and fetal outcomes.

## Introduction

Hypertensive disorders in pregnancy, particularly pregnancy-induced hypertension (PIH), are among the leading causes of maternal and fetal morbidity and mortality worldwide. PIH refers to the development of new-onset hypertension after 20 weeks of gestation and is classified into a spectrum of conditions, including gestational hypertension, pre-eclampsia, and eclampsia [1]. These conditions affect approximately 5%-10% of all pregnancies globally and are responsible for a significant portion of maternal deaths, particularly in low- and middle-income countries where access to adequate prenatal care may be limited [2]. The incidence of PIH has increased in recent years, correlating with the rise in maternal obesity, advanced maternal age, and higher rates of assisted reproductive technologies [3].

Pre-eclampsia, a more severe form of PIH, is characterised by hypertension and proteinuria after 20 weeks of pregnancy, often accompanied by signs of end-organ damage, such as renal impairment, liver dysfunction, and thrombocytopenia [4]. If untreated, pre-eclampsia can progress to eclampsia, which is marked by the occurrence of seizures and can lead to life-threatening complications, including placental abruption, stroke, multiorgan failure, and fetal death [5]. The underlying pathophysiology of pre-eclampsia and eclampsia is complex and not fully understood, but key factors include endothelial dysfunction, placental ischemia, immune dysregulation, and oxidative stress [6,7]. Moreover, the development of these conditions is influenced by genetic, environmental, and immunological factors [8].

A critical aspect of the pathophysiology of PIH is its impact on the coagulation system. Pregnant women with PIH often experience abnormalities in their coagulation profiles, including thrombocytopenia, increased platelet activation, and alterations in the coagulation cascade [9]. These changes can result in a hypercoagulable state, predisposing women to complications such as disseminated intravascular coagulation (DIC), haemorrhage, and thromboembolic events [10]. Studies have demonstrated that coagulation markers, including platelet count, prothrombin time (PT), activated partial thromboplastin time (aPTT), and fibrinogen levels, are significantly altered in women with pre-eclampsia and eclampsia [11]. Thrombocytopenia, in particular, is considered one of the earliest haematological changes observed in pre-eclampsia and is used as a prognostic indicator of disease severity [12].

The evaluation of coagulation parameters and haematological profiles in PIH patients is essential for the early detection of potential complications and the implementation of timely interventions [13]. For instance, monitoring platelet count can help identify thrombocytopenia associated with increased risks of maternal haemorrhage during delivery. Similarly, assessing PT, aPTT, and bleeding time can provide insights into the extent of coagulation abnormalities, guiding clinical decisions regarding anticoagulation therapy and the timing of delivery [14]. Understanding the coagulation dynamics in women with PIH is particularly important in the context of severe pre-eclampsia and eclampsia, where the risk of coagulopathy-related complications is heightened [6].

Despite the wealth of research on PIH, there remains a lack of consensus regarding the predictive value of specific coagulation and haematological markers in determining adverse maternal and fetal outcomes [15]. While some studies have identified significant correlations between abnormal coagulation parameters and the severity of hypertensive disorders, others have found these associations to be inconsistent [16]. Additionally, variations in laboratory methodologies, sample populations, and clinical management practices contribute to the difficulty in establishing standardised diagnostic and therapeutic protocols.

Given these challenges, this study compares the coagulation profile and haematological parameters in women with PIH and normotensive pregnant women. By identifying key differences between these groups, the study seeks to enhance the understanding of the haematological and coagulation abnormalities associated with hypertensive disorders of pregnancy. The findings of this study may contribute to the development of improved diagnostic tools and management strategies for women at risk of PIH-related complications.

## Materials and methods

Study design

This study was a cross-sectional, observational study conducted over two years, from June 2022 to June 2024, in the Department of Pathology at Jawaharlal Nehru Medical College (JNMC), Sawangi (Meghe), in collaboration with the Department of Obstetrics and Gynaecology at Acharya Vinoba Bhave Rural Hospital (AVBRH), Sawangi, Wardha, Maharashtra, India. The Institutional Ethics Committee approved the study and all participants provided informed consent after being educated about the trial’s specifics.

Study population, inclusion criteria and exclusion criteria

The study included pregnant women attending routine antenatal check-ups at AVBRH. Participants were categorised into two groups: those with pregnancy-induced hypertension (PIH) and normotensive pregnant women. The inclusion criteria for the PIH group included gestational hypertension (systolic blood pressure >140/90 mmHg after 20 weeks of gestation, with no proteinuria and BP returning to normal post-delivery), mild to severe pre-eclampsia, and eclampsia (characterised by convulsions not attributable to other causes). Normotensive pregnant women with no history of hypertension were included as controls. Patients with pre-existing medical conditions, such as diabetes mellitus, renal diseases, thyroid disorders, chronic hypertension, severe anaemia, autoimmune disorders, or those receiving anticoagulant therapies (e.g., aspirin), were excluded. Additionally, patients with multifetal gestation, HELLP (haemolysis, elevated liver enzymes, low platelet count) syndrome, or sepsis were not included in the study. Smokers and those who did not consent to participate were also excluded.

Sample size determination

The sample size was calculated using Cochran's formula for sample size estimation in observational studies. With a significance level of 5% (95% confidence interval), the proportion of patients with PIH (p) estimated at 10%, and a margin of error (e) set at 8%, the total sample size was calculated to be 55 patients with PIH and 55 normotensive pregnant women, making a total of 110 participants.

Data collection

Blood samples were collected from all participants using a 23-gauge needle under strict aseptic conditions to ensure contamination-free venipuncture. After cleaning the venipuncture site with an alcohol swab, a tourniquet was applied to the upper arm, and blood was drawn from a prominent vein. Care was taken to minimise hemolysis during sample collection. Approximately 5 ml of blood was collected from each participant. The collected blood samples were then divided into two different types of vacutainers. The samples were transferred into ethylenediaminetetraacetic acid (EDTA) vials to analyse haematological parameters, including platelet count. As an anticoagulant, EDTA helped preserve the blood sample for accurate platelet analysis. For the coagulation profile, blood was transferred into vials containing 3.2% sodium citrate, which chelates calcium ions to prevent clotting during transport and storage. To maintain the integrity of the samples, all blood vials were stored at room temperature and transported to the laboratory within one hour of collection. The coagulation parameters, such as bleeding time (BT), clotting time (CT), prothrombin time (PT), and activated partial thromboplastin time (aPTT), were promptly assessed within one to three hours after collection to avoid any time-dependent alterations in the results. Platelet counts and related indices, such as mean platelet volume (MPV) and platelet distribution width (PDW), were also analysed during this timeframe.

Laboratory procedures

Platelet Count Estimation

The venous blood collected in EDTA vials was thoroughly mixed with gentle inversion to ensure the even distribution of anticoagulants throughout the sample. The samples were then analyzed using a fully automated haematology analyser (Beckman Coulter, Brea, CA) [17]. The analyzer was calibrated before use, and whole blood mode was selected to ensure accurate platelet count measurements. The instrument was set to “Ready” mode, and the sample tubes were inverted several times before being attached to the sample probe. Once the start button was pressed, the system aspirated the sample and displayed the platelet count within 60 seconds. Any abnormal findings or flagged results were noted for further investigation.

Prothrombin Time (PT) Estimation

Prothrombin time was measured to assess the extrinsic pathway of coagulation. The blood collected in sodium citrate vials was centrifuged for PT testing to separate plasma from the blood cells. After centrifugation, 50 µl of plasma was transferred into a cuvette preloaded into an automated coagulation analyser. Thromborel S, the reagent for PT testing, was added in 100 µl increments to the plasma sample. The reaction was initiated, and the machine automatically calculated the time taken for clot formation. The results were expressed in seconds, and the machine flagged abnormal PT values for additional review.

Activated Partial Thromboplastin Time (aPTT) Estimation

The aPTT was measured to evaluate the intrinsic pathway of coagulation. Like the PT test, the plasma was obtained by centrifuging the blood collected in sodium citrate vials. A 50 µl of plasma was added to a cuvette and placed in the automated coagulation analyser. The aPTT reagent, consisting of activated cephaloplastin and calcium chloride, was sequentially added in two stages. First, 50 µl of the cephaloplastin reagent was introduced into the plasma sample, and after a designated wait time, 50 µl of calcium chloride was added to initiate the clotting reaction. The analyser automatically calculated the time taken for clot formation, which was recorded as the aPTT value.

Bleeding Time (BT) Estimation

Bleeding time was measured using Duke’s method [18]. A standardised skin puncture was made using a lancet, typically on the fingertip or earlobe, and the time taken for bleeding to stop was recorded. The filter paper was used to blot the blood every 30 seconds until the bleeding ceased. The bleeding time was then calculated and recorded.

Clotting Time (CT) Estimation

Clotting time was assessed using Wright’s capillary tube method [19]. A small quantity of blood was drawn into a capillary tube and held horizontally. The time taken for the blood to clot inside the tube was recorded. After regular intervals, the tube was gently broken to assess the formation of a fibrin thread, indicating clot formation. All test results were recorded and compiled for statistical analysis, with mean values, standard deviations, and significant differences between groups assessed using appropriate statistical methods.

Statistical analysis

Quantitative data were summarised using means and standard deviations, while qualitative data were expressed as proportions. Comparative analyses between the case and control groups were conducted using the student’s t-test and chi-square test. A p-value of ≤ 0.05 was considered statistically significant. Statistical analyses were performed using SPSS Version 27.0 and GraphPad Prism Version 7.0.

## Results

Table 1 shows the age distribution of patients in both the case and control groups. Most patients in both groups were aged 18-30 years, with 40 patients (72.73%) in the case group and 50 patients (90.91%) in the control group. A statistically significant difference was observed between the two groups, with the case group having a higher mean age (27.98 ± 4.92) compared to the control group (25.32 ± 3.82) (p = 0.002), indicating that pregnancy-induced hypertension (PIH) may be more prevalent in older pregnant women.

**Table 1:** Age distribution of patients S: Significant, p-value is considered significant (p<0.05)

Age distribution	Case Group		Control Group	
	No. of patients	Percentage	No. of patients	Percentage
18-30	40	72.73	50	90.91
31-40	14	25.45	5	9.09
>40	1	1.82	0	0.00
Total	55	100.00	55	100.00
Mean ± SD	27.98±4.92		25.32±3.82	
P-value	0.002S			

Table 2 compares the prevalence of symptoms between the case and control groups. Swelling of the lower limbs and oedema were present in 55 patients (100%) in the case group. At the same time, these symptoms were rare in the control group, with only three patients (5.45%) showing swelling of the lower limbs and 11 patients (20%) exhibiting oedema. Additionally, symptoms like headaches were present in nine patients (16.36%), visual disturbances in four patients (7.27%), and epigastric pain in three patients (5.45%) in the case group. The statistical analysis reveals significant differences in symptom prevalence, especially for lower limb swelling and edema (p < 0.0001), highlighting their association with PIH.

**Table 2:** Distribution of patients according to symptoms S: Significant, NS: Non-Significant, p-value is considered significant (p<0.05, 0.001)

Symptoms	Case Group		Control Group		P-value
	No. of patients	Percentage (%)	No. of patients	Percentage (%)	
Headache	9	16.36	0	0.00	0.0018S
Swelling of lower limbs	55	100.00	3	5.45	<0.0001S
Visual disturbances	4	7.27	0	0.00	0.04S
Vomiting	7	12.73	13	23.64	0.13NS
Epigastric pain	3	5.45	0	0.00	0.08NS
Convulsions	0	0.00	0	0.00	-
Oedema	55	100.00	11	20.00	<0.0001S

Table 3 shows that the blood pressure levels between the case and control groups differed. The systolic blood pressure (SBP) and diastolic blood pressure (DBP) were significantly higher in the case group (SBP: 143.41 ± 16.5; DBP: 93.01 ± 8.16) compared to the control group (SBP: 116.07 ± 7.4; DBP: 74.18 ± 6.03), with p-values of <0.0001 for both measurements. These findings confirm that elevated blood pressure is a defining feature of pregnancy-induced hypertension.

**Table 3:** Distribution of patients according to blood pressure S: Significant, p-value is considered significant (p<0.05). SBP: Systolic blood pressure; DBP: diastolic blood pressure

BP	Case Group		Control Group		P-value
	Mean	SD	Mean	SD	
SBP	143.41	16.5	116.07	7.4	<0.0001S
DBP	93.01	8.16	74.18	6.03	<0.0001S

Table 4 shows that urine albumin levels were assessed to evaluate proteinuria, a hallmark of pre-eclampsia. In the case group, 32 patients (58.18%) showed 3+ albumin levels, while no proteinuria was detected in the control group. Additionally, 17 patients (30.91%) in the case group had trace albumin levels, three patients (5.45%) had 1+ albumin, and three patients (5.45%) had 2+ albumin. This indicates a significant difference between the groups, with severe proteinuria strongly associated with PIH (p = 0.36 for trace protein and higher).

**Table 4:** Distribution of patients according to urine albumin values NS: Non-Significant, p-value is considered significant (p<0.05)

Urine Albumin	Case Group		Control Group		P-value
	No. of patients	Percentage (%)	No. of patients	Percentage (%)	
Absent	0	0.00	0	0.00	0.36NS
Trace	17	30.91	0	0.00	
1+	3	5.45	0	0.00	
2+	3	5.45	0	0.00	
3+	32	58.18	0	0.00	
Total	55	100.00	0	0.00	

Table 5 shows the clinical diagnosis of the patients. In the case group, pre-eclampsia was the most common diagnosis, with 25 patients having that condition (45.45%), followed by gestational hypertension in 21 patients (38.18%). Severe pre-eclampsia was diagnosed in three patients (5.45%), eclampsia in two patients (3.64%), and pregnancy-induced hypertension (PIH) in four patients (7.27%). There were statistically significant differences between the case and control groups, with the control group consisting entirely of 55 normotensive pregnancies (p < 0.0001), reinforcing the distinction between the two populations.

**Table 5:** Distribution of patients according to clinical diagnosis S: Significant, NS: Non-Significant, p-value is considered significant (p<0.05, 0.001)

Clinical Diagnosis	Case Group		Control Group		P-value
	No. of patients	Percentage (%)	No. of patients	Percentage (%)	
Eclampsia	2	3.64	0	0.00	0.15NS
Pre-eclampsia	25	45.45	0	0.00	<0.0001S
Gestational hypertension	21	38.18	0	0.00	<0.0001S
Pregnancy-induced hypertension	4	7.27	0	0.00	0.04S
Severe pre-eclampsia	3	5.45	0	0.00	0.08S
Normotensive pregnancy	0	0.00	55	100.00	<0.0001S
Total	55	100.00	55	100.00	

Table 6 shows that bleeding time was prolonged in the case group, with 38 patients (69.09%) having bleeding times between 151 and 190 seconds, compared to 31 patients (56.36%) in the control group. The mean bleeding time in the case group was significantly higher (185.76 ± 21.7 seconds) than in the control group (156.72 ± 20.50 seconds), with a p-value of <0.0001. This suggests that PIH may affect coagulation, leading to longer bleeding times.

**Table 6:** Distribution of patients according to bleeding time S: Significant; SD: standard deviation

Bleeding Time (seconds)	Case Group		Control Group	
	No. of patients	Percentage (%)	No. of patients	Percentage (%)
110-150	2	3.64	22	40.00
151-190	38	69.09	31	56.36
191-230	12	21.82	2	3.64
231-260	3	5.45	0	0.00
Total	55	100.00	55	100.00
Mean ± SD	185.76±21.7		156.72±20.50	
P-value	<0.0001S			

Table 7 shows that the platelet count was significantly lower in the case group compared to the control group. In the case group, 34 patients (61.82%) had platelet counts between 1.7 and 2.6 lakh/cu.mm, while in the control group, 28 patients (50.91%) had counts between 2.7 and 3.6 lakh/cu.mm. The mean platelet count in the case group (1.77 ± 0.50 lacs/cu.mm) was markedly lower than in the control group (3.09 ± 0.61 lakh/cu.mm), with a p-value of <0.0001. This finding points to thrombocytopenia as a significant issue in PIH.

**Table 7:** Distribution of patients according to platelet count S: Significant; SD: standard deviation

Platelet count (lacs/cu.mm)	Case Group		Control Group	
	No. of patients	Percentage (%)	No. of patients	Percentage (%)
0.6-1.6	21	38.18	0	0.00
1.7-2.6	34	61.82	14	25.45
2.7-3.6	0	0.00	28	50.91
3.7-4.3	0	0.00	13	23.64
Total	55	100.00	55	100.00
Mean ± SD	1.77±0.50		3.09±0.61	
P-value	<0.0001S			

Table 8 shows that the clotting time was generally shorter in the case group than in the control group. The mean clotting time in the case group was 292.47 ± 54.04 seconds, while in the control group, it was 317.89 ± 73.62 seconds, with a p-value of 0.04. This difference suggests that the coagulation process is accelerated in patients with PIH, potentially increasing the risk of clotting-related complications.

**Table 8:** Distribution of patients according to clotting time S: Significant; SD: standard deviation

Clotting Time (seconds)	Case Group		Control Group	
	No. of patients	Percentage (%)	No. of patients	Percentage (%)
210-260	21	38.18	15	27.27
261-311	13	23.64	10	18.18
312-362	13	23.64	15	27.27
363-413	8	14.55	10	18.18
414-464	0	0.00	2	3.64
465-510	0	0.00	3	5.45
Total	55	100.00	55	100.00
Mean ± SD	292.47±54.04		317.89±73.62	
P-value	0.04S			

Table 9 shows that prothrombin time (PT) was significantly lower in the case group, with 34 patients (61.82%) exhibiting PT values between 8.1 and 10 seconds, while most patients in the control group (29 patients, 52.73%) had values between 10.1 and 12 seconds. The mean PT in the case group was 9.6 ± 1.01 seconds, compared to 11.78 ± 1.01 seconds in the control group (p < 0.0001). This suggests that PIH may lead to faster coagulation times, contributing to an increased risk of clot formation.

**Table 9:** Distribution of patients according to prothrombin time S: Significant; SD: standard deviation

Prothrombin Time (seconds)	Case Group		Control Group	
	No. of patients	Percentage (%)	No. of patients	Percentage (%)
6.1-8	3	5.45	0	0.00
8.1-10	34	61.82	2	3.64
10.1-12	18	32.73	29	52.73
12.1-14	0	0.00	24	43.64
Total	55	100.00	55	100.00
Mean ± SD	9.6±1.01		11.78±1.01	
P-value	<0.0001S			

Table 10 shows the international normalised ratio (INR) was lower in the case group, with a mean INR of 0.8 ± 0.08 compared to 0.98 ± 0.08 in the control group. The difference was statistically significant (p < 0.0001), suggesting altered coagulation dynamics in patients with PIH, which may increase the likelihood of thrombotic events.

**Table 10:** Distribution of patients according to international normalised ratio (INR) S: Significant; SD: standard deviation; p-value is considered significant (p<0.05, 0.001)

Parameter	Case Group		Control Group		P-value
	Mean	SD	Mean	SD	
International normalised ratio (INR)	0.8	0.08	0.98	0.08	<0.0001S

Table 11 shows that activated partial thromboplastin time (aPTT) was lower in the case group, with 30 patients (54.55%) showing aPTT values between 22 and 25 seconds. In contrast, 23 patients (41.82%) in the control group had values between 30 and 33 seconds. The mean aPTT in the case group (26.04 ± 2.97 seconds) was significantly shorter than in the control group (29.5 ± 3.67 seconds), with a p-value of <0.0001, indicating faster coagulation in patients with PIH.

**Table 11:** Distribution of patients according to activated partial thromboplastin time S: Significant; SD: standard deviation

aPTT (sec)	Case Group		Control Group	
	No. of patients	Percentage (%)	No. of patients	Percentage (%)
22-25	30	54.55	13	23.64
26-29	17	30.91	13	23.64
30-33	8	14.55	23	41.82
34-37	0	0.00	6	10.91
Total	55	100.00	55	100.00
Mean ± SD	26.04±2.97		29.5±3.67	
P-value	<0.0001S			

Table 12 shows that in terms of management, most patients in both groups underwent emergency lower segment cesarean sections (EMLSCS). However, a higher percentage of patients in the control group had full-term normal deliveries (FTND). There was no significant difference in management strategies between the two groups (p = 0.44), suggesting that clinical decisions regarding delivery were consistent across both PIH and normotensive patients.

**Table 12:** Distribution of patients according to management NS: Non-Significant, p-value is considered significant (p<0.05, 0.001) EMLSCS: emergency lower segment cesarean sections; FTND: full-term normal deliveries; PTVD: pre-term vaginal delivery; LSCS: lower (uterine) segment caesarean section.

Management	Case Group		Control Group		P-value
	No. of patients	Percentage (%)	No. of patients	Percentage (%)	
EMLSCS	49	89.09	43	78.18	0.44NS
FTND	5	9.09	11	20.00	
PTVD	0	0.00	1	1.82	
LSCS	1	1.82	0	0.00	
Total	55	100.00	55	100.00	

## Discussion

The findings of this study provide significant insights into the haematological and coagulation abnormalities associated with pregnancy-induced hypertension (PIH). As demonstrated, PIH is associated with notable changes in both coagulation dynamics and platelet counts, suggesting that hypertensive disorders of pregnancy can lead to a hypercoagulable state. This is consistent with previous studies that have identified increased risks of thromboembolic complications in women with PIH [20]. The hypercoagulable state observed in the study participants with PIH is characterised by shorter prothrombin time (PT), activated partial thromboplastin time (aPTT), and lower international normalised ratio (INR). These findings align with the current understanding that PIH induces endothelial dysfunction and systemic inflammation, activating the coagulation cascade and contributing to a higher risk of clot formation [2]. Moreover, the study confirms that thrombocytopenia, a key haematological change in PIH, plays a crucial role in the disease's progression. Thrombocytopenia, as demonstrated by significantly reduced platelet counts in women with PIH, has been well-documented as one of the earliest haematological markers of disease severity [21]. The reduction in platelet counts observed in this study suggests that platelet consumption occurs due to endothelial injury, a hallmark of hypertensive disorders of pregnancy [6]. Platelet activation and aggregation are heightened in response to endothelial damage, leading to thrombocytopenia, which can further predispose patients to bleeding complications during labour and delivery [22].

Despite the hypercoagulable state, the prolonged bleeding time in the case group suggests a complex interaction between platelet function and the coagulation system in women with PIH. Although the coagulation process is accelerated, platelet dysfunction appears to impair the hemostatic response, leading to a paradoxical increase in bleeding tendency [23]. This dual risk of thrombosis and bleeding presents a significant challenge in managing PIH, particularly in severe cases like pre-eclampsia and eclampsia, where the balance between preventing clot formation and controlling bleeding becomes critical [24]. The presence of proteinuria in a significant proportion of the case group, particularly in women with pre-eclampsia, reinforces its role as a diagnostic marker. Proteinuria, along with hypertension, remains one of the defining features of pre-eclampsia, indicating renal dysfunction as a result of the systemic endothelial damage [3]. In this study, 58.18% of patients with PIH exhibited 3+ albumin levels, a finding that aligns with other research highlighting the relationship between proteinuria and disease severity in hypertensive disorders of pregnancy [15]. The strong association between proteinuria and PIH underscores the importance of regular monitoring of renal function in women with hypertensive disorders during pregnancy.

In addition to the coagulation and haematological findings, the study highlights the clinical significance of blood pressure control in managing PIH. The elevated systolic and diastolic blood pressure levels in the case group further validate the importance of early detection and management of hypertension in pregnancy [25]. The significant difference in blood pressure between the PIH and normotensive groups emphasises the need for vigilant prenatal monitoring to prevent the progression of PIH into more severe forms, such as pre-eclampsia and eclampsia, which are associated with life-threatening complications such as placental abruption, stroke, and multi-organ failure [26]. Although this study provides important insights, there remains a lack of consensus regarding the predictive value of specific coagulation and haematological markers in determining adverse maternal and fetal outcomes in PIH. Some studies have reported significant correlations between abnormal coagulation parameters and the severity of hypertensive disorders, while others have found inconsistent associations [27]. The variability in results may be attributed to differences in laboratory methodologies, patient populations, and clinical management practices, further complicating the development of standardised diagnostic and therapeutic guidelines [7].

Furthermore, while the study suggests that coagulation abnormalities may be used as markers for predicting disease severity, the clinical utility of these markers in guiding treatment decisions remains uncertain. For instance, the role of prophylactic anticoagulation in women with PIH is still debated, with some studies advocating for its use to prevent thromboembolic events. In contrast, others caution against the increased risk of haemorrhage [28]. Further research is needed to clarify the role of coagulation and haematological markers in the risk stratification and management of PIH. The study also has implications for the timing and mode of delivery in women with PIH. As noted, the majority of patients in both groups underwent emergency lower segment cesarean sections (EMLSCS), indicating that severe PIH and associated coagulation abnormalities often necessitate surgical intervention [29]. However, the decision to perform a cesarean section should be carefully weighed against the risks of haemorrhage, particularly in women with significant thrombocytopenia [30]. While this study did not find significant differences in management strategies between the two groups, the findings underscore the need for individualised management plans based on the patient’s coagulation profile, blood pressure control, and clinical presentation [31].

Limitations

The limitations of this study include a relatively small sample size, which may limit the generalizability of the findings to larger populations. Additionally, the study was conducted at a single centre, potentially introducing selection bias. Variability in clinical management and laboratory methodologies could also affect the consistency of results. Lastly, the study did not assess long-term maternal or neonatal outcomes, which would be valuable in understanding the broader implications of haematological and coagulation abnormalities in pregnancy-induced hypertension.

## Conclusions

In conclusion, this comparative study of coagulation profiles and haematological parameters in pregnancy-induced hypertension (PIH) reveals significant alterations in coagulation dynamics and haematological markers among women with PIH compared to normotensive pregnant women. The study found that women with PIH exhibited a hypercoagulable state characterised by lower prothrombin time (PT), activated partial thromboplastin time (aPTT), and international normalised ratio (INR), along with thrombocytopenia. These abnormalities may contribute to an increased risk of thromboembolic events and haemorrhagic complications, especially during delivery. Additionally, the study demonstrates that platelet count and bleeding time are useful indicators of disease severity in PIH, providing valuable clinical insights for timely intervention. Overall, these findings emphasise the importance of routine monitoring of coagulation and haematological parameters in PIH patients to mitigate the risk of maternal and fetal complications.
